# Ultrasound abnormalities of the major salivary glands in Egyptian patients with systemic sclerosis

**DOI:** 10.1007/s10067-023-06763-w

**Published:** 2023-09-18

**Authors:** Ahmed E. Hafez, AlShaimaa M. Taha, Abdelhfeez Moshrif, Hany M. Aly, Rasha Abdel Noor, Mohamed Mortada, Radwa Elkhouli

**Affiliations:** 1 Department of Rheumatology and Rehabilitation, Faculty of Medicine, Minia University, Minia, Egypt; 2 Department of Biochemistry, Faculty of Science, Ain Shams University, Cairo, Egypt; 3 Department of Rheumatology, Faculty of Medicine, Al-Azhar University, Assiut, Egypt; 4 Rheumatology and Rehabilitation, Faculty of Medicine, Al-Azhar University, Cairo, Egypt; 5 Department of Internal Medicine, Faculty of Medicine, Tanta University, Tanta, Egypt; 6 Department of Rheumatology and Rehabilitation, Zagazig University, Zagazig, Egypt; 7 Department of Rheumatology, Rehabilitation, and Physical Medicine, Faculty of Medicine, Tanta University, Tanta, Egypt

**Keywords:** Parotid, Submandibular, Power Doppler, Inflammatory markers, Association, Schirmer’s test

## Abstract

**Introduction/objectives:**

systemic sclerosis (SSc) is an autoimmune disorder with multiple organs destruction. This study aimed to identify the ultrasonographic changes of major salivary glands in Egyptian scleroderma patients and to detect their association to different disease manifestations.

**Methods:**

Forty-seven SSc patients and 43 apparent healthy volunteers were enrolled. Demographics, inflammatory markers, and autoimmune status were recorded. Ultrasound evaluation of salivary glands was performed. Salivary gland changes’ associations were statistically examined with SSc susceptibility and disease manifestations.

**Results:**

Thirty-one SSc patients exhibited glandular pathology (*p* < 0.0001), compared to controls. Of these abnormalities, SSc patients showed a total parotid gray scale of 2, total submandibular gray scale of 2, total glandular gray scale of 4, and total glandular Doppler signal of 1 at *p* < 0.0001, compared to the control group. Patients with SSc and glandular pathology had a higher prevalence of arthritis (*p* = 0.029) and ESR (*p* = 0.002) than those with normal glandular ultrasound. Significant associations were reported between gray scale ultrasound (GSUS) of total parotid (odds ratio “OR” = 0.4), total submandibular (OR = 0.36), and total glandular (OR = 0.53) with susceptibility to SSc at *p* < 0.0001. Total glandular GSUS (*p* = 0.039) and total submandibular power Doppler (*p* = 0.044) correlated with the SSc duration. Total parotid GSUS (*p* = 0.008) and total glandular GSUS (*p* < 0.0001) correlated with Schirmer’s test.

**Conclusions:**

Major salivary glands are affected in SSc. Hence, scanning these glands with ultrasound is an additive tool besides the current practice.

**Table:** 

**Key Points** • *Major salivary gland changes, observed by ultrasonography, are new findings in Egyptian SSc patients.* • *Ultrasound changes of major salivary glands are associated with inflammatory markers and clinical manifestations of SSc.* • *Scleroderma ultrasonography scans of the main salivary glands could be added to the routine work.*

## Introduction

Systemic sclerosis (SSc) is a multi-organ autoimmune disease in which the pathological inflammatory process, vasculopathy, and fibrosis cause multiple disease manifestations [1]. Typically, it progresses destructively to several organs, including the skin, joints, gastrointestinal system, heart, lungs, and kidneys. In the first 1 or 2 years of the disease, 40% of patients experience organ damage that may be irreversible and affects one or more organs [2]. Sicca symptoms are common in SSc patients. In previous years, few studies reported the involvement of abnormalities in salivary and lacrimal glands in patients with SSc that resulted in hyposalivation and dry eye complaints [3, 4]. Since the publication of Alarcón-Segovia and his colleagues in 1973 who described SSc as the most elusive among all connective tissue diseases. They concluded that minor salivary glands changes are due to collagen deposition, which is related to the pathological process in SSc, and they found that ductal changes are related to autoimmunity. Hence, Sjogren syndrome (SS) was implicated making this disease the most baffling among connective tissue diseases [5]. Several studies followed with conflict results being a natural course of SSc pathogenesis or is relation to associated SS [6–9]. The mechanism of sicca manifestations in SSc is believed to be due to the fibrosis process of the salivary glands (SGs) [10, 11]. SGs represent the target organ for autoimmunity in SS [12]. SS manifests in the exocrine glands resulting in sicca symptoms and other systemic extra-glandular manifestations including cutaneous, musculoskeletal, renal, pulmonary, hematological, and neurological affection [13]. The disease course may be discrete, but it can coexist with autoimmune diseases like rheumatoid arthritis and systemic lupus erythematosus [14, 15]. Secondary Sjogren in systemic sclerosis has been investigated seldom with limited data available, with reported frequencies of 7.5%, 14%, 23%, up to 28% [9, 10, 16–18].

Nowadays, ultrasound (US) scan in rheumatic diseases is an additional clinical skill for many health practitioners that requires long-term training and practice [19]. Ultrasonography of the salivary gland is an emerging tool in recent decades with excellent opportunities and has many advantages [9, 20]. A subtask force of the Outcome Measures in Rheumatology Clinical Trials (OMERACT) working group studied the use of salivary gland ultrasound as a possible outcome instrument for the measurement of salivary gland abnormalities in patients with primary Sjogren syndrome (pSS) [21]. There is no international consensus for defining these abnormalities in patients with SSc.

The aforementioned studies prompted us to investigate whether or not our Egyptian scleroderma patients’ main salivary glands changed, and whether or not these changes correlated with clinical symptoms and signs, inflammatory markers, and autoantibodies.

## Patients and methods

### Patients

This case-control study was conducted at the outpatient clinics of rheumatology from April 2022 to October 2022. Forty-seven Egyptian adult patients with systemic sclerosis were enrolled in the current study. SSc patients were diagnosed according to the American College of Rheumatology (ACR)/European Alliance of Rheumatology Associations (EULAR) [22]. They were classified into limited and diffused systemic sclerosis according to LeRoy’s criteria [23]. Forty-three apparent healthy volunteers were involved and served as a control group. Juvenile cases, pregnant, lactating mothers, individuals with a history or current; hepatitis C and human immunodeficiency viral infection, lymphoma, graft versus host disease, amyloidosis, diabetics, other rheumatic diseases, and those with history of parotitis were excluded. Also, subjects exposed to radiation and individuals who received anticholinergic drugs were excluded. The study was conducted according to the World Medical Association Declaration of Helsinki and was approved by the Institutional Review Board, Faculty of Medicine, Minia University (Serial No. 312-2022). Signed consent was obtained from all participants.

### Clinical and laboratory evaluation

Data collected from all the participants include age, gender, disease duration at the time of presentation, current treatments received, clinical characteristics, cutaneous manifestations, musculoskeletal manifestations, joint symptoms, Raynaud’s phenomenon, digital ulcers or scars, and interstitial lung disease (ILD). Erythrocyte sedimentation rate (ESR) and C-reactive protein (CRP) were measured in all subjects as markers for inflammation. All participants were screened for autoimmune profiling through a check for antinuclear antibody (ANA), rheumatoid factor (RF), anti-Ro60/SSA, anti-La/SSB, anti-topoisomerase 1 (anti-topo I), and anti-centromere (ACA). The presence of sicca symptoms was checked by a standard questionnaire [24]. Schirmer’s test without anesthesia [25] was applied at the same setting of glandular scanning. Finally, all participants were exposed to an assessment of skin tightness using the modified Rodnan total skin score [26].

### Ultrasound examination of salivary gland

Evaluation of the salivary glands was performed by experienced rheumatologists who are EULAR, certified in musculoskeletal US with more than 7 years’ experience at the same sitting for clinical evaluation and laboratory sampling.

Examination was performed according to EULAR standardized procedures for ultrasound imaging in rheumatology [27]. AM and HA used a 14 MHz Toshiba xario-200 from Japan; RA and AH used an 8 MHz to 12 MHz South Korean Albinion Cube 8 Diamond; MM and RE used a 10 MHz to 18 MHz American AH LOGIC E 10 GE. All machines were equipped with power Doppler (PD) detection and common specifications including frequency range of 5–10 MHz, a low–medium wall filter, and a pulse repetition frequency of 400–1000 Hz were commonly adjusted for the involved machines.

The major salivary glands (both parotids and both submandibular) were scanned. Scanning was done by gray scale ultrasound (GSUS) and PD in two planes; longitudinal and transverse. During scanning, patient head was turned opposite to the scanned side to scan the parotids then hyperextended to visualize the submandibular glands while the patient was advised to relax supinated [6]. Regarding GSUS scanning, a semiquantitative 4 grades US was applied according to the OMERACT recommendations [21], each salivary gland was scored on (0–3) scoring system by GSUS [15]. That score depends on glandular inhomogeneity and the presence of hypo/anechoic areas. Normal salivary glands are homogenous echotexture granular pattern in ultrasound comparable to thyroid echogenicity and its visualized as hyperechoic structure when compared to nearby tissues, with frequent normal vasculatures. G0, normal; G1, mild inhomogeneity with no anechoic/hypoechoic areas; G2, moderate inhomogeneity with focal anechoic/hypoechoic areas; G3, diffuse inhomogeneity with anechoic/hypoechoic areas occupying the entire gland surface or fibrous echo-structures. The presence of hyperechoic bands on the gland surface, which evolves into fibrotic tissue, characterizes the fibrous formations. A 0–12 total OMERACT salivary gland ultrasound (SGUS) grade was calculated as the sum of the single grades of the four glands. Each pair of glands was also given a score between zero and six. Intraglandular PD was also graded from zero to 3 according to the parenchymal blood flow. Glands were scored 0 if there is no flow, graded as 1 if there is up to three isolated signals, if the parenchymal flow was seen in < 50% of the echotexture it is G2 and if its > 50% then G3 will be applied [20]. According to previous records [28], a salivary gland GSUS score ≥ 2 was considered pathological. Hence, sub-classification was applied into pathological (if ≥ 2) and nonspecific if (< 2) in one salivary gland (parotid or submandibular).

### Statistical analysis

The power of the study was computed utilizing G^*^Power 3.1.9.7 software (Franz Faul, Universität Düsseldorf, Germany) (https://www.psychologie.hhu.de/arbeitsgruppen/allgemeine-psychologie-und-arbeitspsychologie/gpower). The test family was adjusted at *F* test and the statistical test used was adjusted at ANCOVA: fixed effects, main effect, and interactions. The input parameters were effect size *F* = 0.4, *β*/*α* ratio = 2, total sample size = 90, numerator df = 10, number of groups = 2, and number of covariates = 1. All statistical analyses were performed using the Statistical Package for Social Science version 26 for Windows (SPSS software package, Chicago, USA). The tests of data normality were statistically determined using the Kolmogorov–Smirnov test with Lilliefors significance correction. The categorical data were expressed as frequencies (percentages) and nonparametric data were expressed as median (interquartile range). The nonparametric data were examined between the SSc group and control group using Mann–Whitney statistical analysis. *χ*^2^ test was utilized to assess the differences in the categorial data between SSc patients and the control individuals.

Binary logistic regression analysis was operated to conduct the power of the association of salivary gland abnormalities with the susceptibility to SSc disease compared to the control subjects. The power of associations was quantified by the odds ratio (OR) with a 95% confidence interval (CI) adjusted for age and gender. Multiple linear regression analysis was used to investigate the association of the independent variables relative to the dependent variables after transforming nonparametric variables into the logistic scale. The regression models were established using the “enter” analysis. All *p* values were 2-sided, and a *p* value < 0.05 was considered statistically significant. For association analyses, the statistical significance thresholds were set to *p* < 0.0125 after Bonferroni correction.

## Results

The demographics of all participants are listed in Table 1. The control subjects were age and sex matched chosen to be matched in age with the SSc group (*P* value = 0.665). The frequency of male and female gender was 9.3 and 90.7%, respectively, in the control group. On the other hand, the frequency was 12.80% for males and 87.20% for females among SSc patients. Patients had SSc disease for a median of 48 months. A percentage of 17.00 of SSc patients had a limited cutaneous SSc disease, while 83.00% had a diffused cutaneous SSc. Among SSc patients, 61.70% showed xerostomia, 59.60% had xerophthalmia, 85.11% showed sicca symptoms in the form of dry eye and/or dry mouth, 70.20% had arthritis, 74.50% had interstitial lung disease (ILD), and 48.90% had fingers ulcer/scars, compared to the control group (*p* value < 0.0001). Furthermore, all SSc patients had Raynaud’s phenomenon (100%). Of SSc patients, 38.30% had a pathological Schirmer’s test (< 5 mm, *p* < 0.0001), compared to the control group. SSc patients showed a high skin score with a median of 22. In addition, SSc patients received different treatments either alone or in combination; 24 patients received colchicine therapy, 25, 21, 13, and 4 patients treated with steroid, methotrexate, cyclophosphamide, and mycophenolate mofetil, respectively (*p* value < 0.0001).

**Table 1:** Demographic characteristics of patients with SSc, compared to the control group

	Control (*n* = 43)	SSc patients (*n* = 47)	*χ*^2^ value	*p* value
Age (years)	41.00 (18)	39.00 (13)		0.665
Gender *n* (%)				
Male	4 (9.30)	6 (12.80)	0.273	0.601
Female	39 (90.70)	41 (87.20)		
Duration of SSc disease (months)	0 (0)	48.00 (69)		** < 0.0001**
SSc type *n* (%)				
Limited		8 (17.00)	90.00	** < 0.0001**
Diffused		39 (83.00)		
Xerostomia *n* (%)				
No	38 (88.40)	18 (38.30)	23.95	** < 0.0001**
Yes	5 (11.60)	29 (61.70)		
Xerophthalmia *n* (%)				
No	40 (93.00)	19 (40.40)	27.51	** < 0.0001**
Yes	3 (7.00)	28 (59.60)		
Sicca *n* (%)				
Negative	37 (86.00)	7 (14.90)	45.50	** < 0.0001**
Positive	6 (14.00)	40 (85.10)		
Raynaud’s phenomenon *n* (%)				
No	41 (95.30)	0 (0)	82.31	** < 0.0001**
Yes	2 (4.70)	47 (100)		
Arthritis *n* (%)				
Absent	43 (100)	14 (29.80)	47.67	** < 0.0001**
Present	0 (0)	33 (70.20)		
ILD *n* (%)				
Absent	43 (100)	12 (25.50)	52.40	** < 0.0001**
Present	0 (0)	35 (74.50)		
Fingers/scar ulcer *n* (%)				
No	43 (100)	24 (51.10)	28.27	** < 0.0001**
Yes	0 (0)	23 (48.90)		
Schirmer’s test (mm)	20.00 (10)	4.00 (8)		** < 0.0001**
Pathological Schirmer’s test *n* (%)				
Normal (> 5 mm)	43 (100)	29 (61.70)	20.59	** < 0.0001**
Pathologic (< 5 mm)	0 (0)	18 (38.30)		
Skin score	0 (0)	22.00 (15.00)		** < 0.0001**
Colchicine treatment (yes/no)	0/43	24/23	28.27	** < 0.0001**
Steroid treatment (yes/no)	0/43	25/22	31.67	** < 0.0001**
Methotrexate (yes/no)	0/43	21/26	25.06	** < 0.0001**
Cyclophosphamide treatment (yes/no)	0/43	13/34	13.90	** < 0.0001**
Mycophenolate mofetil treatment (yes/no)	0/43	4/43	3.83	0.05

Data are expressed as median (interquartile range) for nonparametric variables and frequencies (percentages) for categorical variables

*SSc* systemic sclerosis, *ILD* interstitial lung disease

The mean difference is significant at *p* < 0.05 using asymptotic significance 2-sided

Clinical and laboratory features of SSc patients, compared to the control subjects, are represented in Table 2. There were significant elevations in the inflammatory markers (ESR and CRP) among SSc patients, compared to the control individuals (*p* < 0.0001). Moreover, 74.50% of SSc patients were positive for ANA test with high titer (*p* < 0.0001), compared to the control group. Only 34 and 10.60% of SSc patients showed positive results for anti-topo I (*p* < 0.0001) and ACA (*p* = 0.028) antibodies, respectively, compared to the control group. Regarding anti-Ro and anti-La antibodies, 17.00 and 14.90% among SSc patients showed positive anti-Ro (*p* = 0.005) and anti-La (*p* = 0.008) tests, respectively, compared to the control group. Of SSc patients, 78.70% showed negative results for both anti-Ro and anti-La tests, while 21.30% were positive for any of them (*p* < 0.0001).

**Table 2:** Clinical and laboratory investigations in patients with SSc, compared to the control group

	Control (*n* = 43)	SSc Patients (*n* = 47)	χ^2^ value	*p* value
ESR (mm/h)	18.00 (13.00)	34.00 (27.00)		** < 0.0001**
CRP (mg/ L)	0 (0)	12.00 (18.00)		** < 0.0001**
ANA titer	0 (0)	160.00 (240.00)		** < 0.0001**
ANA *n* (%)				
Negative	40 (93.00)	12 (25.50)	41.93	** < 0.0001**
Positive	3 (7.00)	35 (74.50)		
Anti-topo I *n* (%)				
Negative	43 (100)	31 (66.00)	17.80	** < 0.0001**
Positive	0 (0)	16 (34.00)		
RF *n* (%)				
Negative	38 (88.40)	38 (80.90)	0.97	0.325
Positive	5 (11.60)	9 (19.1)		
ACA *n* (%)				
Negative	43 (100)	42 (89.40)	4.84	**0.028**
Positive	0 (0)	5 (10.60)		
Anti-Ro *n* (%)				
Negative	43 (100)	39 (83.0)	8.03	**0.005**
Positive	0 (0)	8 (17.00)		
Anti-La *n* (%)				
Negative	43 (100)	40 (85.10)	6.94	**0.008**
Positive	0 (0)	7 (14.90)		
Combined anti-Ro and anti-La *n* (%)				
Negative for Both	43 (100)	37 (78.70)	90.00	** < 0.0001**
Positive for Any	0 (0)	10 (21.30)		

Data are expressed as median (interquartile range) for nonparametric variables and frequencies (percentages) for categorical variables

*SSc* systemic sclerosis, *ESR* erythrocyte sedimentation rate, *CRP* C-reactive protein, *ANA* antinuclear antibody, *anti-topo I* anti-topoisomerase I, *RF* rheumatoid factor, *ACA* anti-centromere antibody, *Anti-Ro* anti-SSA, *Anti-La* anti-SSB

The mean difference is significant at *p* < 0.05 using asymptotic significance 2-sided

**Fig. 1:**
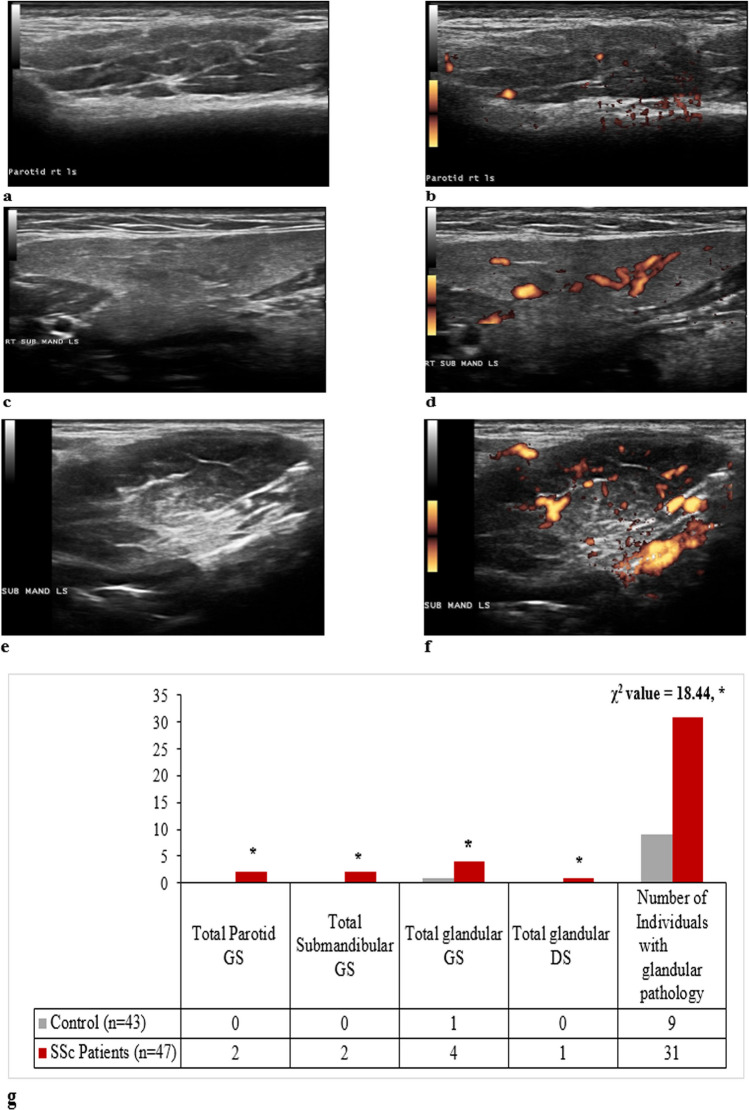
Representative ultrasound images showing four-grade semiquantitative scoring system of salivary gland. **a** GSUS of the parotid (grade 2). **b** PDUS of the parotid gland (grade 1). **c** GSUS changes of the submandibular gland (grade 1). **d** PDUS of the submandibular gland (grade 2). **e** GSUS of the submandibular gland (grade 3). **f** PDUS of the submandibular gland (grade 3). **g** Data are expressed as median (interquartile range) for nonparametric variables and frequencies (percentages) for categorical variables. SSc, systemic sclerosis; GS, gray scale; DS, Doppler signal; GSUS, gray scale ultrasound; PDUS, power Doppler ultrasound. A single asterisk (*) denotes significance at *P* < 0.0001, compared to the control individuals. The mean difference is significant at *p* < 0.05 using asymptotic significance 2-sided

The changes in the homogeneity of the salivary gland, observed by the ultrasound in the studied groups, are represented in Fig. 1a–f. These changes were observed by 6 observers with strong inter-rater Kappa agreement of 0.79–0.86. Among the SSc group, 31 patients exhibited glandular pathology (*p* < 0.0001), compared to the control individuals. Of these abnormalities, SSc patients showed a total parotid gray scale of 2, total submandibular gray scale of 2, total glandular gray scale of 4, and total glandular Doppler signal of 1 at *p* < 0.0001, compared to the control group (Fig. 1g).

Data shown in Table 3 illustrates the difference between SSc patients with and without glandular pathology regarding the clinical and laboratory features. There were no statistical differences in all clinical and laboratory features between SSc patients with and without the glandular pathology except for the presence of arthritis and ESR values. Of SSc patients, 80.60% with glandular pathology had arthritis (*p* = 0.029) in association with having a higher ESR value (50%, *p* = 0.002) than those with normal glandular US.

**Table 3:** Clinical and laboratory features of SSc patients with and without glandular pathology

	SSc patients Without glandular pathology (*n* = 16)	SSc patients With glandular pathology (*n* = 31)	*χ*^2^ value	*p* value
Schirmer’s test (mm)	4.50 (8)	3.00 (8.00)		0.709
Pathological Schirmer’s test *n* (%)				
Normal (> 5 mm)	9 (56.30)	20 (64.50)	0.31	0.581
Pathologic (< 5 mm)	7 (42.80)	11 (35.50)		
Skin score	20.00 (21.25)	24.00 (16.00)		0.135
Duration of SSc disease (months)	60 (48.00)	48 (90.00)		0.521
Xerostomia *n* (%)				
No	6 (37.50)	12 (38.70)	0.01	0.936
Yes	10 (62.50)	19 (51.30)		
Xerophthalmia *n* (%)				
No	7 (43.80)	12 (38.70)	0.11	0.739
Yes	9 (56.30)	19 (61.30)		
Sicca *n* (%)				
Negative	2 (12.50)	5 (16.10)	0.11	0.741
Positive	14 (87.50)	26 (83.90)		
Raynaud’s phenomenon *n* (%)				
No	0 (0)	0 (0)		
Yes	16 (100.00)	31 (100)		
Arthritis *n* (%)				
Absent	8 (50.00)	6 (19.40)	4.74	**0.029**
Present	8 (50.00)	25 (80.60)		
ILD *n* (%)				
Absent	5 (31.30)	7 (22.60)	0.42	0.518
Present	11 (68.80)	24 (77.40)		
Fingers ulcer *n* (%)				
No	10 (62.50)	14 (45.20)	1.27	0.260
Yes	6 (37.50)	17 (54.80)		
ESR (mm/ hr.)	30.00 (13)	45 (31)		**0.002**
CRP (mg/ L)	6.00 (11.00)	12.00 (18.00)		0.114
ANA titer	160.00 (220.00)	160 (240)		0.420
ANA *n* (%)				
Negative	3 (18.80)	9 (20.0)	0.59	0.444
Positive	13 (81.30)	22 (71.00)		
Anti-topo I *n* (%)				
Negative	10 (62.50)	21 (67.70)	0.13	0.719
Positive	6 (37.50)	10 (32.30)		
RF *n* (%)				
Negative	13 (81.30)	25 (80.60)	0.002	0.960
Positive	3 (18.80)	6 (19.40)		
ACA *n* (%)				
Negative	13 (81.30)	29 (93.50)	1.68	0.195
Positive	3 (18.80)	2 (6.50)		
Anti-Ro *n* (%)				
Negative	12 (75.00)	27 (87.10)	1.09	0.296
Positive	4 (25.00)	4 (12.90)		
Anti-La *n* (%)				
Negative	11 (68.80)	26 (83.90)	1.44	0.230
Positive	5 (31.30)	5 (16.10)		

Data are expressed as median (interquartile range) for nonparametric variables and frequencies (percentages) for categorical variables

*SSc* systemic sclerosis, *ESR* erythrocyte sedimentation rate, *CRP* C-reactive protein, *ANA* antinuclear antibody, *anti-topo I* anti-topoisomerase I, *RF* rheumatoid factor, *ACA* anti-centromere antibody, *Anti-Ro* anti-SSA, *Anti-La* anti-SSB

The mean difference is significant at *p* < 0.05 using asymptotic significance 2-sided

Results in Table 4 showed significant associations of salivary gland abnormalities (total parotid GS “OR = 0.40, 95% CI = 0.25–0.64,” total submandibular GS “OR = 0.36, 95% CI = 0.22–0.60,” and total glandular GS “OR = 0.53, 95% CI = 0.39–0.73”) with the risk of SSc, compared with the control group, at* p* value < 0.0125 after the Bonferroni correction. However, the total glandular Doppler signal did not show a significant association with the risk of SSc, compared to the control group (*p* value = 0.994).

**Table 4:** Association of salivary gland abnormalities with SSc using binary logistic regression

	SSc vs. Control	
	^#^Adjusted OR (95% CI)	*p* value
Total parotid GS	0.40 (0.25–0.64)	** < 0.0001**
Total submandibular GS	0.36 (0.22–0.60)	** < 0.0001**
Total glandular GS	0.53 (0.39–0.73)	** < 0.0001**
Total glandular PD	0.0 (0.0–0.0)	0.994

*SSc* systemic sclerosis, *GS* gray scale, *PD* power Doppler, *OR* odds ratio, *95% CI* 95% confidence interval

^#^Adjusted for age and gender

*p* < 0.0125 was considered significant after the Bonferroni correction

Table 5 describes the importance of the observed salivary gland abnormalities as independent variables for SSc duration, Schirmer’s test, skin score, and ANA titer as dependent variables among all individuals. The salivary gland abnormities showed significant correlations only with SSc duration and Schirmer’s test. Both total glandular GS (*β* = 0.37, *t* = 2.16 at *p* = 0.039) and total submandibular PD (*β* =  − 0.38, *t* =  − 2.12 at *p* = 0.044) showed significant correlations with the SSc duration. On the other hand, both total parotid GS (*β* =  − 0.43, *t* =  − 2.83 at *p* = 0.008) and total glandular GS (*β* =  − 0.50, *t* =  − 4.15 at *p* < 0.0001) showed highly significant correlations with Schirmer’s test.

**Table 5:** Multiple linear regression analysis for the association of salivary gland abnormalities with the common diagnostic markers of SSc

Dependent Variable	Predictors	*R*	*r*^2^	*F*	*p* value^*^	*β*	*t*	*p* value	VIF
Duration of SSc disease	Total parotid GS	0.08	0.01	0.16	0.70	0.08	0.40	0.695	1
	Total submandibular GS	0.24	0.06	1.75	0.20	0.24	1.32	0.20	1
	Total glandular GS	0.37	0.14	4.68	0.039	0.37	2.16	**0.039**	1
	Total glandular PD	0.38	0.14	4.48	0.044	− 0.38	− 2.12	**0.044**	1
Schirmer’s test	Total parotid GS	0.43	0.18	8.02	0.008	− 0.43	− 2.83	**0.008**	1
	Total submandibular GS	0.31	0.10	4.08	0.05	− 0.31	− 2.02	0.05	1
	Total glandular GS	0.50	0.25	17.19	< 0.0001	− 0.50	− 4.15	** < 0.0001**	1
	Total glandular PD	0.14	0.02	0.55	0.461	0.14	0.74	0.461	1
Skin score	Total parotid GS	0.10	0.01	0.25	0.622	0.10	0.50	0.622	1
	Total submandibular GS	0.08	0.01	0.20	0.662	0.08	0.44	0.662	1
	Total glandular GS	0.13	0.02	0.47	0.50	0.13	0.69	0.498	1
	Total glandular PD	0.16	0.03	0.70	0.411	0.16	0.83	0.411	1
ANA titer	Total parotid GS	0.10	0.01	0.23	0.634	− 0.10	− 0.48	0.636	1
	Total submandibular GS	0.16	0.03	0.75	0.395	0.16	0.86	0.395	1
	Total glandular GS	0.18	0.03	0.98	0.330	0.18	0.99	0.330	1
	Total glandular PD	0.11	0.01	0.28	0.603	0.11	0.53	0.603	1

*SSc* systemic sclerosis, *β* standardized coefficient, *VIF* variance inflation factor, *GS* gray scale, *PD* power Doppler, *ANA* antinuclear antibody

^*^p value obtained from ANOVA table

*p* < 0.05 was considered significant

## Discussion

Scleroderma is a systemic autoimmune disease characterized by extensive fibrosis, vasculopathy together with abundant autoantibodies. Vasculopathy is the initial process in scleroderma that affects almost all organs leading to ischemic changes. Progressive fibrosis gradually disrupts skin and internal organ architecture and leads to organ dysfunction [29]. The hyposecretory function of salivary glands is well documented in several autoimmune diseases; Sjogren’s syndrome, rheumatoid arthritis, autoimmune thyroiditis, systemic lupus erythematosus, type 1 *diabetes mellitus*, primary biliary cirrhosis, and chronic graft versus host disease with different explanatory theories [30]. The current study was set to record ultrasound abnormalities in the major salivary glands of Egyptian patients with SSc and to detect if there is an association between these changes and different disease manifestations.

The current study illustrated a high prevalence of Egyptian SSc patients suffering from sicca symptoms. In addition, they had positive Schirmer’s test in a significant number of them. All of which agree with the previous reports [6, 11, 31]. Furthermore, SSc patients were characterized by the elevations in the inflammatory markers (ESR and CRP) which is in line with Lakota et al. [32]. In agreement with previous studies [6], the autoimmunity status expressed by ANA, anti-topo I, ACA, anti-Ro, and anti-La positivity was recorded among SSc patients.

In addition to the typical symptoms of SSc, such as interstitial lung disease, Raynaud’s phenomenon, arthritis, fingers ulcer/scars, and sicca symptoms, two-thirds of our patients also displayed novel ultrasound findings, including salivary glandular alterations in echogenicity and enhanced PD signals in the glandular echotexture which are also recently observed in the SSc patients [6, 9]. Regarding the clinical and laboratory manifestations, SSc patients with glandular pathology differ from those with normal glandular features only in having arthritis with higher ESR values. This might explain that the incidence of severe inflammation among SSc patients can be related to the incidence of salivary gland abnormalities observed among these patients. Previous studies showed that ESR is increased in SSc and predicts mortality [33, 34]. ESR is one of the parameters incorporating the modified Medsger SSc disease severity scale [35, 36]. The occurrence of a systemic inflammatory process in SSc patients had been reported [32] due to elevating levels of circulating inflammatory cytokines and chemokines which are associated with internal organ involvement and allow the discrimination between limited and diffuse forms of SSc [37]. These cytokines and chemokines are also reported to be related to the incidence of salivary gland abnormalities [38].

As far as we know, no reports statistically analyzed the association between salivary gland abnormalities and susceptibility to SSc disease. The current study reports a strong association of total parotid GS, total submandibular GS, and total glandular GSUS with the existence of the disease. To the best of our knowledge, there are no previous studies that have reported the relationship between salivary gland US abnormalities and different disease parameters in SSc patients. A close association was reported in the current study between both total glandular GSUS and total submandibular PD on one side and the SSc duration on the other side. Also, associations of both total parotid GSUS and total glandular GSUS were recorded with the Schirmer’s test.

## Conclusion

In conclusion, major salivary glands ultrasound changes observed by the gray scale and the power Doppler in Egyptian patients with SSc are exciting new findings and linked to disease susceptibility, various clinical signs and symptoms, and inflammatory markers. These results emphasize the value of ultrasound as a quick, non-invasive bedside investigation to recognize significant abnormalities of the salivary glands in SSc patients. Future studies could be performed on a larger sample of SSc patients with limited and diffuse subtypes. Different cytokines and chemokines may be evaluated in SSc patients with and without salivary gland abnormalities. Moreover, other studies on other autoimmune diseases could be conducted to differentiate these changes in Egyptian patients.

## Data Availability

Data are available under reasonable request to the corresponding author.
